# Gas chromatography-mass spectrometry based serum metabolic analysis for premature infants and the relationship with necrotizing enterocolitis: a cross-sectional study

**DOI:** 10.1186/s13052-019-0646-6

**Published:** 2019-04-29

**Authors:** Fusheng Wang, Weizhong Li, Guanghuan Wang, Menglu Yu, Jun Zhong, Chenbin Xu, Danli Li, Yongcui Zhou

**Affiliations:** 1 Department of Pediatric Surgery, The Second Affiliated Hospital of Shantou University Medical College, Shantou, 515041 China; 2 Department of Neonatal, The Second Affiliated Hospital of Shantou University Medical College, Shantou, 515041 China; 3 Reproductive Medicine Centre, The First Affiliated Hospital of Shantou University Medical College, Guangdong Province, Shantou, 515041 China

**Keywords:** Premature infants, Serum, Metabonomic, Necrotizing enterocolitis

## Abstract

**Background:**

Preterm birth and feeding are the most important pathogenic factors of neonatal necrotizing enterocolitis (NEC). Metabonomic has been widely used in the diagnosis and treatment of other diseases, but there is no research on the related diseases of premature infants. Compared with full-term infants, the metabolism of preterm infants has its own specificity, so it can easily lead to NEC and other digestive tract inflammatory diseases. Metabonomic may be applied to the diagnosis of preterm related diseases, such as NEC.

**Methods:**

The model was established with premature infant serum samples from 19 premature infants in our hospital, which was compared with the full-term infant control group. Serum was analyzed by gas chromatography-mass spectrometry (GC-MS), coupled with the analysis of serum metabolic characteristics. The variable important in projection, *P* value and Pearson correlation coefficient of samples were analyzed by using SIMCA, SPSS and other multivariate statistical analysis software.

**Results:**

Compared to the term infants, premature infants had significantly higher levels of luteolin, and lower levels of xylose, O-succinyl-L-homoserine and lauric acid in the serum. There were some correlations among several different metabolites and clinically related indices (albumin, total bilirubin) for premature birth related diseases.

**Conclusions:**

There are metabolic alterations in the serum of premature infants, which make contribution to the diagnosis of NEC.

## Background

With the improvement of the level of economy and the accuracy of clinical diagnosis and treatment, the survival of premature infants is increasing [1]. However, due to the immature development of organs and systems in preterm infants, metabolism is more susceptible to the internal and external environment, which leads to the occurrence of various systemic diseases [2]. Among them, NEC is one of the most serious diseases. Premature delivery is one of the leading risk factors for NEC [3]. Among NEC patients, preterm infants account for 75–95% [4]. In addition, preterm infants have higher mortality, as well as worse prognosis, from NEC than term infants [5]. Although treatment of NEC is improving, its mortality remain high. Therefore, it is particularly important to explore the etiology, pathogenesis and early prevention of NEC. However, there is no specific index that can predict the occurrence of NEC at the early stage. The purpose of this study is to collect serum from non-fed preterm infants, then use gas chromatography-mass spectrometry to analyze the metabolic profile and explore the metabolic differences between preterm and term infants. The study uses universal screening to select the metabolites closely related to NEC, because the results have not been validated.

## Methods

### Clinical data

Nineteen patients were recruited from those born in our hospital during June 2017 to January 2018 (birth month 29^+4^week–34^+3^week, birth weight 1.5–2.1 kg). We enrolled all newborns meeting the inclusion criteria. Bell’s stage of NEC was used to make NEC diagnosis (Bell’s stage ≥ 1). The physicians diagnosing NEC were blinded to the results of the GC-MS detection of serum. An informed, written parental consent was obtained, and the study protocol was approved by the ethics committee of our hospital.

Inclusion criteria: (1) Gestational age < 35 weeks; (2) Birth weight < 2.2 kg; (3) No genetic metabolic diseases or congenital malformations, stable vital signs after birth.

Exclusion criteria: Feeding immediately after birth or severe illness.

### GC-MS detection of serum

Collected 3 ml serum before feeding respectively. Samples were removed and thawed. 80 μL of each sample was transferred to a 1.5 ml EP tube and mixed with 10 μL internal standard (L-2-chlorphenylalanine, 0.3 mg/ml) for 10 s. Then, 240 μL methanol: acetonitrile (2:1) was added and mixed with a vortex mixer for 1 min, ultrasonicated under ice water bath for 5 min, and allowed to stand for 10 min at − 20 °C. Samples were centrifuged for 10 min (12,000 rpm, 4 °C), then 150 μL supernatant was loaded into a glass derivative bottle. A quality control sample was prepared by mixing all the sample extracts in equal volume. 80 μL methoxyamine hydrochloride pyridine solution was added to the glass derivative bottle and the oximation reaction was carried out in the shock incubator after 2 min turbulence, then 80 μL BSTFA derivative reagent and 20 μL hexane it was added. After 2 min turbulence, the sample was placed at 70 °C for 60 min, and then GC-MS metabolomics analysis was performed [6].

A 7890B-5977A GC/MSD GC-MS was used to collect data from the US Agilent Co. GC-MS chromatographic conditions involved a DB-5MS capillary column, high purity helium carrier gas, flow rate of 1 ml/min, and inlet 260 °C, injection volume 1 μL. Temperature programming involved heating at 8 °C/min to 125 °C, heating at 5 °C/min to 210 °C, heating at 10 °C/min to 270 °C, and heating at 20 °C/min to 305 °C, then maintaining at 305 °C for 5 min. Mass spectrometric conditions were electron bombardment ion source(EI), ion source 230 °C, four bar 150 °C, and electron energy 70 eV. The scanning mode was full scan mode (SCAN) and the quality scanning range was m/z 50–500. A QC sample was inserted into every 15 analysis samples to examine the repeatability of the whole analysis process.

### Data analysis

After converting the original data of GC/MS into ChemStation (E.02.02.1431) analysis software to CDF format, ChromaTOF 4.34 software was used to preprocess the data for peak extraction, noise elimination, and deconvolution, and the Fiehn database was used to identify the metabolites and to align the peaks, and the three-dimensional data matrix of the CSV format was derived [7].

The internal standard was used for data quality control. The internal standard peaks of the original data matrix and any known false positive peaks were removed, and missing values were replaced by 0. In each sample, all peak signal intensities were normalized. After that, the data was multiplied by 10,000 to remove the redundancy and merge the peaks to get the data matrix. The number of substances detected was 397.

The value of the data matrix was converted into SIMCA14.0 after log2 conversion, and the unsupervised principal component analysis (PCA) was used to observe the overall distribution of the samples and the stability of the entire analysis process. The model was deemed reliable if R^2^X value was greater than 0.5,and then the supervised orthogonal partial least squares analysis (OPLS-DA) was used to differentiate the overall difference of metabolic profiles between groups. In order to prevent the model from over fitting, the quality of the model was investigated by using 7-fold cross validation and 200 response permutation testing (RPT).

The combination of multidimensional analysis and single dimensional analysis was used to screen the differential metabolites between groups. The criteria for screening were a variable important in projection (VIP) > 1 of the first principal component of the OPLS-DA model, and *p*-value of the Student’s t-test < 0.5. Then we calculated the fold change (FC) of the differential metabolites in the two groups.

By MBROLE pathway analysis, the differential metabolites were analyzed, based on the KEGG database (https://www.genome.jp/kegg/pathway.htm), to enrich for relevant metabolic pathways.

From the clinical test results, we selected some index that might be closely related to the differential metabolites, and used the Pearson correlation coefficient in SPSS 21.0 to identify those with high diagnostic value for NEC. The receiver operating characteristic (ROC) curve was plotted, the area under ROC curve (AUC) was calculated, and the diagnostic value for premature birth related diseases of the significant differential metabolites was analyzed.

## Results

### Analysis of serum metabolic profiles of term and preterm infants before feeding

**Fig. 1:**
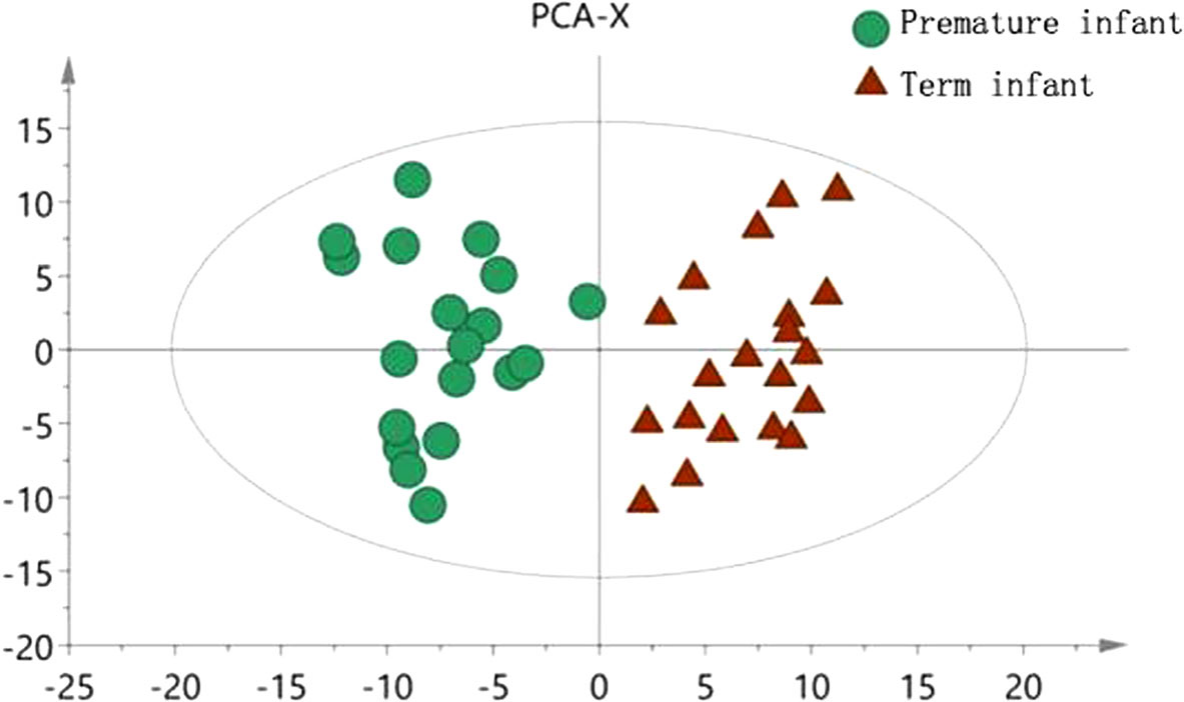
PCA scatter plot in term and preterm groups

**Fig. 2:**
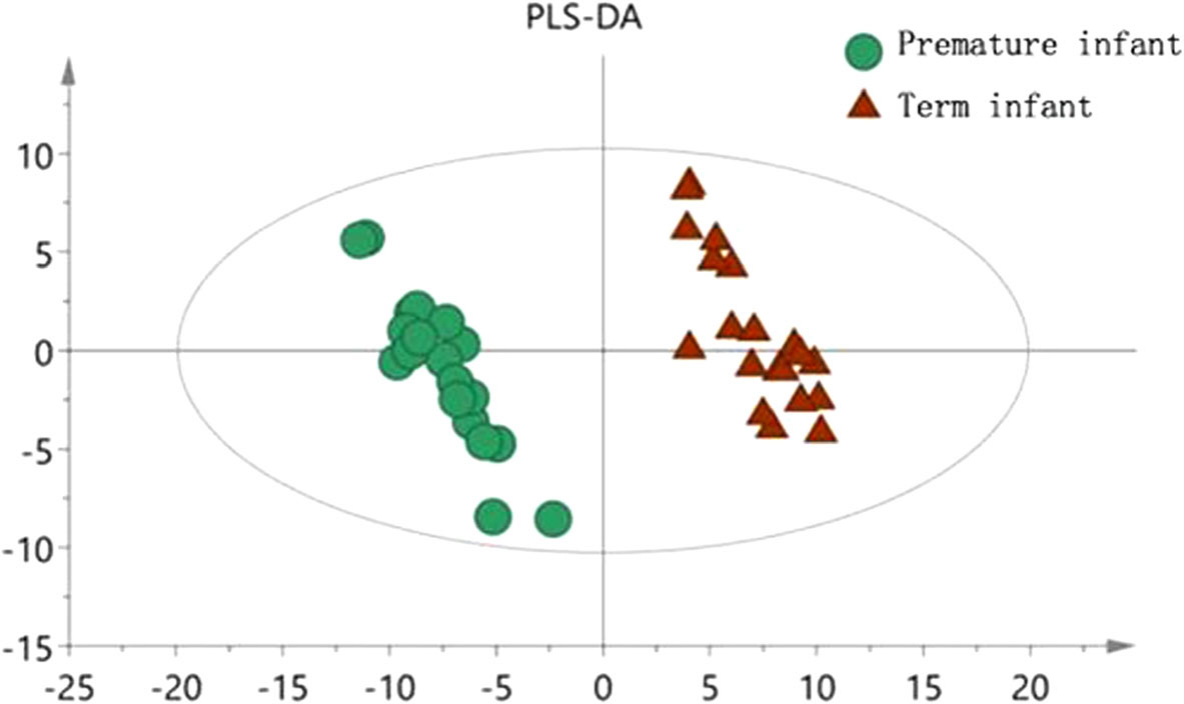
PLS-DA scatter plot in term and preterm groups

**Fig. 3:**
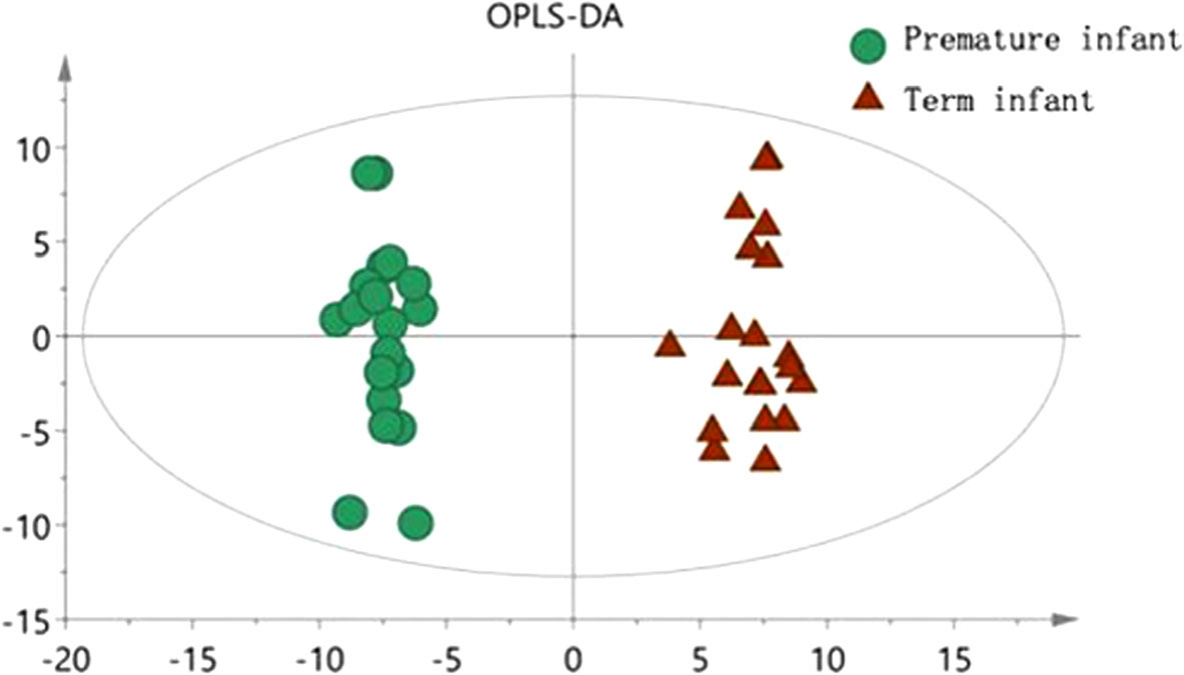
OPLS-DA scatter plot in term and preterm groups

**Fig. 4:**
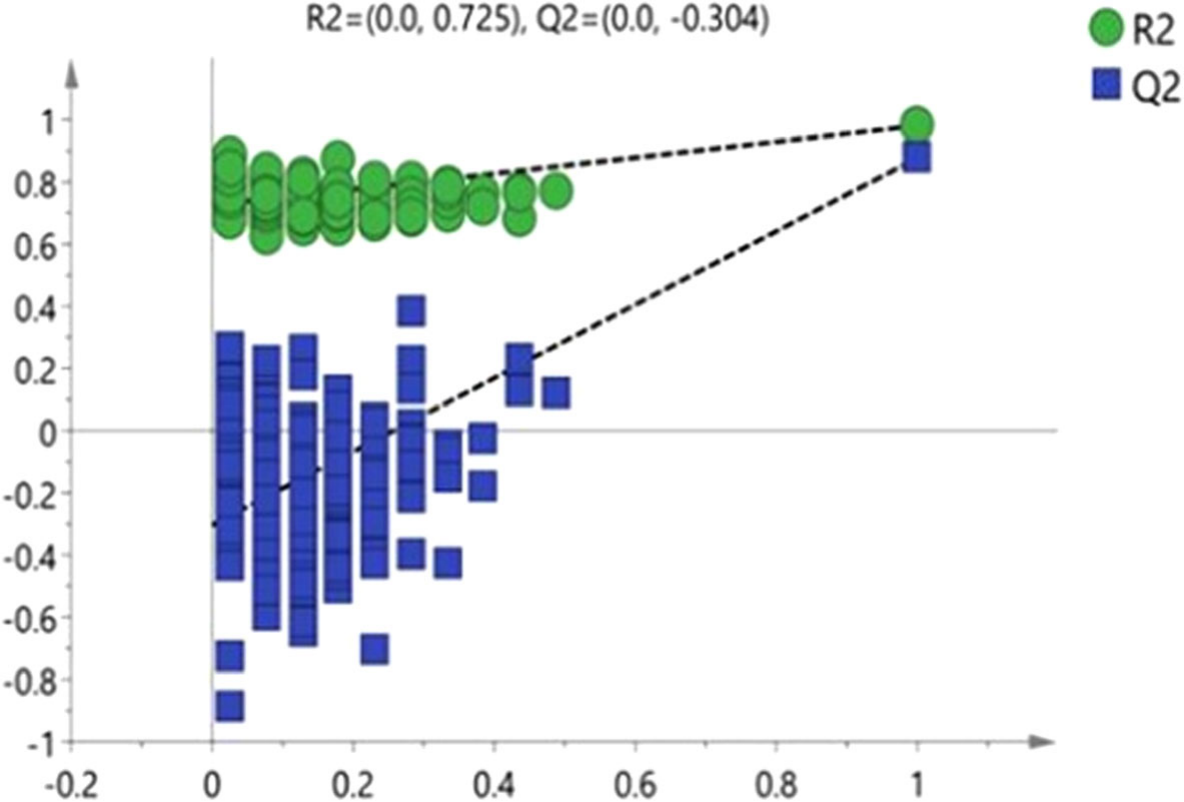
Permutation testing of OPLS-DA in term and preterm groups

The scores for PCA, PLS-DA, OPLS-DA and permutation analysis of the two groups are shown in Figs. 1, 2 and 3, and the result of the 200 response permutation testing of the OPLS-DA model are shown in Fig. 4.

The OPLS-DA model shows that the metabolic profile of preterm infants before feeding is distinctly separated from the term infants group. The R^2^X, R^2^Y and Q^2^ values of the model are 0.602, 0.980 and 0.877 respectively. After 200 replacement tests, the R^2^ and Q^2^ intercept are 0.725 and − 0.304, respectively, suggesting that the OPLS-DA model has not been over fitted, and the two groups have significant differences on the OPLS-DA score map (spectral separation).

### Analysis of serum metabolites and metabolic pathways in term and preterm infants before feeding

In the multidimensional model, 9 metabolites of VIP > 2 were found: xylose, lauric acid, luteolin, gallic acid, O-succinyl-L-homoserine, D-fructose1, 6-bisphosphate, cystine,fructose-6-phosphate, and O-phosphonothreonine. In the single dimensional model, the 9 metabolites found to have the smallest *P* values (*P* < 0.01) were xylose, O-succinyl-L-homoserine, 4-androsten-11β-ol-3,17-dione, analyte 431, gallic acid, O-phosphonothreonine, monostearin, citraconic acid, and D-fructose 1,6-bisphosphate. The results of the screening are shown in Table 1.

**Table 1:** Differential metabolites between premature infants and term infants before feeding

Metabolites	VIP	*P*-value	FC(term infant/premature infant)
xylose	2.646	0.000 E-10	9.077E-7
O-succinyl-L-homoserine	2.251	9.000E-10	1.794E-5
4-androsten-11β-ol-3,17-dione	1.940	1.000E-9	1.498E-1
analyte 431	1.605	1.100E-9	1.335E-1
gallic acid	2.293	3.400E-9	2.976
O-phosphonothreonine	2.014	5.300E-9	1.795
monostearin	1.112	2.020 E-8	3.273
citraconic acid	1.525	2.470 E-8	7.140
D-fructose 1,6-bisphosphate	2.218	5.810 E-8	9.656
lauric acid	2.516	1.586 E-5	2.277E-7
luteolin	2.389	1.556E-5	7.186E4
cystine	2.118	1.074 E-7	2.423 E-1
fructose-6-phosphate	2.102	2.280 E-7	1.770 E-1

**Fig. 5:**
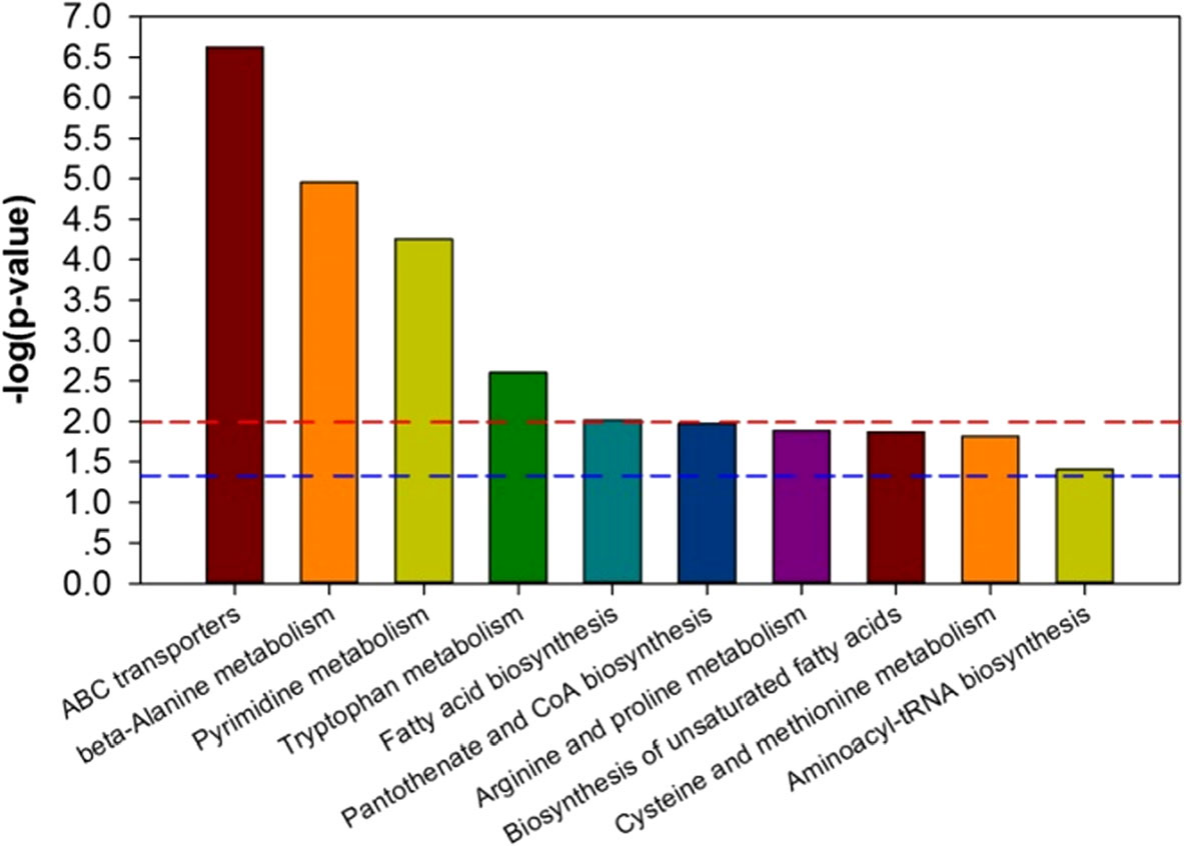
Metabolic pathway enrichment maps in term and preterm groups

Taking metabolic pathway names for the abscissa, and -log (*p*-value) as the ordinate, we mapped the metabolic pathways of preterm/full-term infants before feeding (Fig. 5). A corresponding -log (*p*-value) greater than 2 (*p*-value less than 0.01) means the pathway is significantly different. These metabolites suggest that the metabolic pathways that are disturbed in preterm infants include ABC transporters, β-alanine metabolism, pyrimidine metabolism, tryptophan metabolism, fatty acid biosynthesis, pantothenate and CoA biosynthesis, arginine and proline metabolism, biosynthesis of unsaturated fatty acids, cysteine and methionine metabolism, and aminoacyl-tRNA biosynthesis.

### Correlation analysis between differential metabolites and clinically related indeces

The Pearson correlation coefficients between differential metabolites and clinically related indexes are shown in Table 2. There was a moderate correlation (0.8 > r > 0.5) between albumin and O-succinyl-L-homoserine, citraconic acid, cystine, total bilirubin and xylose, 4-androsten-11β-ol-3,17-dione, analyte 431, gallic acid, monostearin, and lauric acid.

**Table 2:** Pearson correlation coefficients between differential metabolites and clinically related indices

Index	WBC (10^9^/L)	RBC (10^12^/L)	PLT (10^9^/L)	ALB(g/L)	T-Bil (μmol/L)	AST (U/L)	Cr (μmol/L)	CRP (mg/L)	APTT (s)
Metabolite									
xylose	–	–	–	0.451**	0.733**	–	–	–	–
O-succinyl-L-homoserine	–	–	–	0.585**	0.489**	–	–	–	−0.405*
4-androsten-11β-ol-3,17-dione	–	–	–	0.429**	0.565**	–	–	–	–
analyte 431	–	–	–	0.458**	0.714**	–	–	–	–
gallic acid	–	–	–	−0.344*	−0.563**	–	–	–	–
O-phosphonothreonine	–	–	–	−0.372*	−0.469**	–	–	− 0.365*	–
monostearin	–	–	–	−0.397*	− 0.557**	–	–	–	–
citraconic acid	−0.359*	–	–	− 0.502**	−0.497**	–	–	–	–
D-fructose 1,6-bisphosphate	–	–	–	–	−0.496**	–	–	−0.322*	–
lauric acid	–	–	–	0.430**	0.618**	–	–	–	–
luteolin	–	–	–	–	−0.464**	–	–	–	–
cystine	–	–	–	0.501**	0.677**	–	–	0.333*	–
fructose-6-phosphate	–	–	–	0.464**	0.475**	–	–	0.355*	–

**p < 0.01, *p < 0.05, −*p* > 0.05

### Analysis of diagnostic value for premature birth related diseases of differential metabolites

The ROC curve for the differential metabolites and corresponding AUC in Table 3, showed O-phosphonothreonine, luteolin, gallic acid, monostearin, citraconic acid and D-fructose 1,6-bisphosphate to have a high diagnostic value for premature infants with an AUC > 0.9.

**Table 3:** The AUC value for premature birth related diseases of the differential metabolites

Metabolite	Area
xylose	0.000
O-succinyl-L-homoserine	0.075
4-androsten-11β-ol-3,17-dione	0.021
analyte 431	0.011
gallic acid	0.984
O-phosphonothreonine	0.953
monostearin	0.939
citraconic acid	0.984
D-fructose 1,6-bisphosphate	0.925
lauric acid	0.025
luteolin	0.947
cystine	0.042
fructose-6-phosphate	0.116

## Discussion

After birth, premature infants should quickly adapt to the external environment. Because of undeveloped systems, immature state of lipid metabolism, and insufficient reserve of glycogen and fat in the body, premature infants are easily affected by the internal and external environment, which can lead to changes in endocrine hormones in the body. At the same time, the activity of some enzymes stays low or are completely lacking, which also affects the metabolism of corresponding amino acids [8–10]. However, research on the metabolic characteristics of premature infants is still needed. If we can comprehensively identify the metabolic characteristics of premature infants, it will enhance our understanding of the metabolic characteristics of diseases closely related to preterm delivery. Metabonomics is the study of all the metabolites in a cell at a certain time, and metabolites represent the environmental conditions [11]. In this study, we chose the serum of preterm infants as the test object, mainly because changes of serum metabolism could reflect the metabolic characteristics of disease or pathology.

In this study, we found that specific metabolites has clearly changed, among which, luteolin increased, while xylose, O-succinyl-L-homoserine and lauric acid decreased. In clinical follow up, 5 cases were diagnosed as NEC (4 premature infants and 1 full-term infant). The serum metabolites of these cases were analyzed for further discussion. It was found that the differences of gluconic acid, lauric acid and palmitic acid levels were consistent with the research by De Magistris [12] and Wilcock [13], which suggested a possible research direction of NEC metabonomics. Considering the relatively small number of cases, we carried out the correlation analysis between serum metabolic characteristics of premature infants in the study all together, but not NEC infants individually. We will continue to collect clinical data and study the mechanism of metabolite-related diseases.

Metabolic pathway enrichment maps can fully reflect the characteristics of serum metabolites in preterm infants at a given time. In this study, the most significantly changed metabolic pathways involve ABC transporters, β-alanine metabolism, pyrimidine metabolism, tryptophan metabolism, fatty acid biosynthesis, pantothenate and CoA biosynthesis, arginine and proline metabolism, biosynthesis of unsaturated fatty acids, cysteine and methionine metabolism, and aminoacyl-tRNA biosynthesis. It is expected to provide a new way for early prediction of premature delivery relative diseases [14], especially the early prediction of NEC, by exploring the links between multiple metabolites, and the association of proteins, cytokines and other metabolites through a multidimensional model. We can even prevent the occurrence of NEC by interfering with certain metabolic pathways. At present, some people are making their efforts by developing animal models [15].

Pearson correlation analysis, of differential metabolites and clinical test indicators, suggests that the changes in metabolites are related to the changes of clinical indicators. The ROC and AUC analyses showed that certain metabolites, including O-phosphonothreonine, luteolin, gallic acid, monostearin, citraconic acid, and D-fructose 1,6-bisphosphate, have certain diagnostic potential towards premature birth related diseases and NEC. Stewart [16] and Dessì [17], in their previous study, suggested that a single metabolite cannot be used to diagnose NEC, suggesting that multiple metabolites of NEC may be required to diagnose NEC. C-reactive protein (CRP) and calcitonin (PCT) have been used as possible markers for NEC prediction [18].However, we find that the correlation between inflammatory markers and differential metabolites is not significant, which may exclude these indicators as candidate markers, or at least, their relationship should be further confirmed.

## Conclusions

The GC-MS screening of the metabolites of premature infants in this study, to some extent, suggests the potential of the metabolites of preterm infants in the diagnosis of NEC, providing a direction for the exploration of early diagnostic markers for NEC.As our research only screened metabolites, further research is needed for reliable sensitivity and specificity of NEC prediction.

## Abbreviations

AUC: Area under receiver operating characteristic
CRP: C-reactive protein
FC: Fold change
GC-MS: Gas chromatography-mass spectrometry
NEC: Necrotizing enterocolitis
OPLS-DA: Orthogonal partial least squares analysis
PCA: Principal component analysis
ROC: Receiver operating characteristic
RPT: Response permutation testing
VIP: Variable important in projection
